# Genetics, its role in preventing the pandemic of coronary artery disease

**DOI:** 10.1002/clc.23627

**Published:** 2021-05-25

**Authors:** Robert Roberts, Jacques Fair

**Affiliations:** ^1^ College of Medicine, Phoenix, St. Joseph's Hospital and Medical Center The University of Arizona Phoenix Arizona USA

**Keywords:** coronary artery disease, genetics, genetic prediction

## Abstract

Epidemiologists have claimed for decades that about 50% of predisposition for coronary artery disease (CAD) is genetic. Advances in technology made possible the discovery of hundreds of genetic risk variants predisposing to CAD. Multiple clinical trials have shown that cardiac events can be prevented by drugs to lower plasma low‐density lipoprotein cholesterol (LDL‐C). A major barrier to primary prevention is the lack of markers to identify those individuals at risk prior to the development of symptoms of the disease. Conventional risk factors are age‐dependent, occurring mostly in the sixth or seventh decade, which is less than desirable for early primary prevention. A polygenic risk score, derived from the number of genetic risk variants predisposing to CAD inherited by an individual, has been evaluated in over 1 million individuals. The risk for CAD is stratified into high, intermediate, and low. Polygenic risk scores derived from retrospective genotyping of several clinical trials evaluating the effect of statin therapy or PCSK9 inhibitors show the genetic risk is reduced 40%–50% by decreasing plasma LDL‐C. Prospective randomized placebo‐controlled clinical trials document a 40%–50% reduction in cardiac events in individuals at high genetic risk associated with favorable lifestyle changes and increased physical activity. The polygenic risk score is not age‐dependent and remains the same throughout life. Thus, the GRS is superior to conventional risk factors in identifying asymptomatic individuals at risk for CAD early in life for primary prevention. These results indicate clinical embracement of the GRS in primary prevention would be a paradigm shift in the treatment of the number one killer, CAD.

## 1 INTRODUCTION

Advances in technology starting with the sequencing of the human genome in 2001^1^ have enabled the discovery of multiple DNA variants that predispose to multiple diseases. Most common diseases have a significant genetic predisposition which could not be explored until the recent advances made possible by HapMap.^2^ The availability of Single Nucleotide Polymorphisms (SNP's) to provide DNA markers spanning the genome led to the widespread application of Genome‐Wide Association Studies. The present review will summarize these genetic discoveries and how they can be utilized for the early prediction of disease risk. This study will focus on coronary artery disease (CAD). Coronary artery disease has been known for some time to be preventable and clinical trials assessing drugs that lower plasma low‐density lipoprotein cholesterol (LDL‐C) have consistently shown a significant reduction in cardiac events. Secondary prevention of CAD is very successful. Primary prevention of CAD promises to be more rewarding. A major barrier to primary prevention has been the lack of markers to identify those individuals at risk prior to the development of symptoms of the disease. The inadequacy of using conventional risk factors for primary prevention of CAD will be discussed. The role of predicting genetic risk for CAD will be discussed and how it enables primary prevention to be implemented early in life in males and females. Results of randomized, placebo‐controlled clinical trials will be summarized that show genetic risk for CAD can be significantly decreased by drugs that lower the plasma LDL‐C and by favorable changes in lifestyle.

**Hereditary and heart disease**

Epidemiologists have claimed for years that predisposition for CAD is about 50% due to hereditary causes and the remainder being acquired or due to unfavorable lifestyle.^3^ The powerful influence of hereditary factors is displayed in the Utah study in which 14% of the population have a family history of heart disease and in this cohort, 72% of all premature myocardial infarctions and 48% of all coronary events occur.^4^

**The human genome**

**FIGURE 1:**
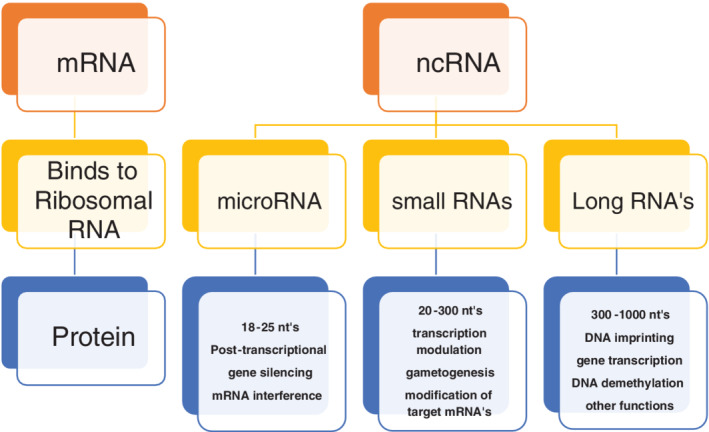
mRNA versus non‐coding RNA. About 1% of DNA codes for mRNA a process refered to as transcription. The mRNA is transported from the nucleus and binds to ribosomal RNA in the cytoplasm. The mRNA provides the template which directs the amino acids to be assembled into proteins a process referred to as translation. Non‐coding RNA is far more common and by definition do not code for protein. These non‐coding RNA's vary in size as indicated and exert their influence directly or indirectly by affecting mRNA's that code for protein

Each strand of the double helix of the human genome contains 3.2 billion bases. The sequencing of the human genome has provided us with a very different functional overview. It is well established that only about 1% of the human genome is involved with sequences that provide the template for proteins. For decades it was assumed that most of the genome was functionless. Today, the opposite picture has emerged with most of the genome providing RNAs that do not code for protein. Nevertheless, these RNAs, which are very promiscuous, can exert their effect anywhere on the chromosome of origin and other chromosomes. The phenotype of an individual is primarily determined by the protein expressed. These non‐coding RNAs influence which proteins are expressed and to what extent. One common mechanism is for non‐coding RNA to bind to the 3′ end of the mRNA, which determines its stability and ultimately translation into protein. However, non‐coding RNA, based on the number of nucleotides, are divided into three categories (micro, intermediate, and long) and have many functions as illustrated in Figure 1. Many human DNA sequences have their origin in simple life forms that originated about 4 billion years ago. It is not surprising that as one moves up the chain into rodents and mammals, their DNA shows considerable overlap with the human DNA sequence. In the interval of 7 million years during which humans and chimpanzee's evolved along separate pathways, their DNA sequences differed by approximately 4%.^5^ The difference in genomes among the *Homo sapiens* is less than 1%.^6^ The sequences that contribute to this difference are primarily large structural variants whose function in large part remains to be determined.^2^ The sequences that contribute most to the unique features of each individual human are primarily single nucleotide polymorphisms.^16^ The number of SNP's per genome is fairly constant at about 5 million.^6^ Furthermore, these SNPs are fairly evenly distributed throughout the genome. Evolution and environmental adaptation are made possible by the new mutations that evolve per generation. These mutations are induced by copy errors^7^ made in the replication of DNA. The DNA molecule is renewed every few days and its fidelity is maintained by complementary base pairing. This mechanism is very precise and makes about one error per billion bases generated, nevertheless, copy errors occur and such errors occurring in germline cells can be transmitted to the next generation. It is expected that most of these errors would be in the form of single nucleotides since the DNA is synthesized one nucleotide at a time. Of the errors, 96% are single nucleotide polymorphisms, another 2% are duplets or triplets, and the remaining 2% might be several nucleotides.^7^ Thus, it is not surprising that over 80% of the unique features of each individual such as the color of one's skin, including predisposition to disease, are due to these SNP's.^8^ These mutations are primarily responsible for the evolution and environmental adaptation. It has been determined that each individual of each new generation inherits about 60 novel mutations.^7^, ^9^ Given the world's population of approximately 8 billion that amounts to 480 billion new mutations.

**Genetic risk variants for polygenic diseases**

Rapid advances have been made starting in the 80s with the discovery of genes responsible for single‐gene disorders, often referred to as Mendelian disorders. These disorders, such as familial cardiomyopathies are rare, occurring in less than 1% of the population, and are highly penetrant with a single gene predominating in the expression of the phenotype.^10^ It required only a few hundred DNA markers to genotype pedigrees of two or three generations. Utilizing genetic linkage analysis it was possible to localize the chromosomal locus of the responsible gene and through cloning and sequencing of the region, identify the precise gene and its mutation.

In contrast, polygenic disorders are due to multiple genes, each of which contributes only minimally to the phenotype. These disorders are very common and the phenotype is significantly influenced by environmental and lifestyle factors. It was recognized very early that genetic linkage analysis would not be the appropriate approach to pursue polygenic disorders. Several advances occurred, making it possible to pursue the genetic architecture of polygenic diseases, such as CAD. The initial discovery was the sequencing of the human genome^11^ followed by annotation of millions of single nucleotide polymorphisms by HapMap.^2^, ^12^ These SNPs provided DNA markers that were distributed throughout the genome. It was now possible to take an unbiased approach utilizing the Case‐Controlled Association Study. Genotyping was performed with SNPs as markers distributed throughout the genome, referred to as Genome‐Wide Association Studies (GWAS).^13^ Rapid genotyping techniques made it possible to genotype large populations of cases and controls with millions of SNPs. The millions of markers utilized required statistical correction to the conventional p‐value of 0.05. There was general agreement that a Bonferroni corrected *p*‐value of 10^−8^ would be adopted, referred to as Genome‐Wide significant risk.^14^ In addition, those markers reaching a *p*‐value of 10^−8^ were required to be replicated in an independent population.

**Discovery of genetic risk variants for CAD**

The application of GWAS led to the simultaneous discovery of 9p21 as the first genetic risk variant for CAD by two independent groups.^15^, ^16^ Features of the 9p21 risk variant confirmed our initial hypothesis that there would be many risk variants contributing to CAD and they would be occurring commonly in the population with each having a minimal risk for CAD. Both groups showed the 9p21 risk variant was associated with only a 25% increased relative risk per copy and occurred in 75% of the world's population. Of considerable interest was the observation that the 9p21 risk variant mediated its risk for CAD independent of all known conventional risk factors. The discovery of the 9p21 risk variant utilized a sample size of 23 000 cases and controls and the Icelandic group utilized a sample size of over 17 000 cases and controls. The 9p21 risk variant was subsequently confirmed by the Welcome Trust Group with a sample size of over 14 000 cases and controls.^17^ While these initial studies were primarily performed in individuals of European descent, subsequent studies confirmed the 9p21 risk variant predisposed to CAD across many other ethnic groups.^18^

The features associated with the 9p21 as a risk variant for CAD confirmed the necessity of having even larger sample sizes in the pursuit of genetic variants predisposing to CAD. This led to the largest international cardiovascular collaboration, referred to as Coronary Artery Disease Genome‐wide Replication And Meta‐Analysis (CARDIoGRAM)^19^ and subsequently as CARDIoGRAMPlusC4D which played a major leadership role in the pursuit of DNA variants predisposing to CAD. In the decade since the discovery of the 9p21 risk variant, the architecture of genetic predisposition for CAD has been unfolding rapidly as documented in a recent review.^18^ The international effort along with many individual groups has discovered 173 genetic risk variants^20^, ^21^ that satisfy genome‐wide significance and have been replicated in an independent population.

**Genetic risk variants as targets for novel drug development**

The future management, whether it be in the form of prevention or treatment, is likely to change dramatically based in part on the genetic risk variants for CAD. These specific attributes have been summarized in a recent review.^20^ Over half of the genetic risk variants mediate their risk for CAD independent of known conventional risk factors for CAD. This implies that while our current emphasis for prevention and treatment focuses on plasma cholesterol, it is self‐evident that many other factors contribute to the pathogenesis of CAD which are yet to be discovered. The Discovery of the pathways by which these risk variants exert and mediate their risk will enable new approaches to this pandemic disease. Secondly, over 80% of the genetic risk variants predisposing to CAD occur in regions of the genome that do not code for protein. This adds further complexity to determine their precise function, but it also directs our research efforts to pursue mechanisms outside protein‐coding sequences. These variants act by modifying the expression of proteins upstream or downstream from the risk variant loci and also are known to do so even on other chromosomes. Thirdly, these variants are very common with over 50% of them occurring in over 50% of the population. The burden of risk for CAD relates to the total number of variants rather than the particular risk associated with an individual variant. Lastly, as predicted, each individual variant exerts only minimal risk for CAD.

**Genetic risk for CAD can be summarized in a single number**

The individual predisposition for CAD is proportional to the number of inherited genetic risk variants by that individual. The total burden of risk is reflected by the accumulative number of risk variants inherited. The number of copies of a genetic risk variant for CAD inherited by an individual will vary from none (no copy in either parent) to one copy in one parent (parent heterozygous) to two parents (parents homozygous). The total CAD risk score is determined by the summation of the number of copies of the risk variant, multiplied by their derived odds ratio.

**Polygenic risk score is superior to conventional risk factors in risk stratification for primary prevention of CAD**

Clinical events due to coronary artery disease have consistently been shown to be preventable. Secondary prevention, such as drugs that lower plasma cholesterol, has been consistently associated with a 30%–40% reduction in relative risk.^22^ A similar approach to primary prevention has also been shown to be very effective.^23^ Coronary artery disease, due to coronary atherosclerosis, develops early and slowly progresses. It usually does not reach a clinical threshold until the fifth or sixth decade, with the incidence of myocardial infarction peaking in males at age 58 and in females at age 68. A limiting factor to primary prevention is identifying those at risk for CAD before the development of symptoms.

Conventional risk factors such as hypertension or diabetes are age‐dependent and usually not present until the sixth or seventh decade of life. The National Lipid Association has recently^24^ summarized the potential role of coronary artery calcium scoring which is also age‐dependent. In contrast to other conventional risk factors, plasma LDL‐C increases early in life and is associated with doubling the risk for CAD for each additional decade of exposure^25^ Ference et al have shown that primary prevention is more effective when initiated early as opposed to later in life.^26^ The genetic risk for CAD is randomly determined at conception thus it is not age‐dependent and can be determined at any time after birth. An example would be a 40‐year‐old male or female with only one risk factor such as plasma LDL‐C of 160 mg/dl which according to current guidelines of the AHA, the ACC, and the ESC would not qualify for any form of preventative therapy. If one is entertaining primary prevention, this would be close to the ideal candidate for primary prevention. One solution is to treat everyone with increased plasma LDL‐C, however, since the average plasma LDL‐C of a 40‐year‐old Westerner is almost twice the recommended level, this would require treating just about everyone. It is also an epidemiological observation^27^ that only about 50% of these individuals, even if they live a normal lifespan, will experience a cardiac event. It is most desirable that we select from among the 50% that is at risk for a coronary event. The use of the polygenic risk score as a complement to the conventional risk factors to identify those individuals at risk will be discussed.

**Retrospective evaluation of the polygenic risk score in clinical trials assessing the effect of statins and PCSK9 inhibitors on cardiac events**

The initial evaluation of the polygenic risk score to predict those at risk of CAD was performed in 2012 utilizing only 12 genetic risk variants.^28^ The results showed only a slight improvement over that of traditional risk factors. A subsequent study was performed^29^ utilizing 27 genetic risk variants and a sample size of 48 421. This randomized, placebo‐controlled study had been performed to assess the effect of statin therapy on cardiac events and involved the combination of 4 well‐known clinical trials. These trials included Justification for the Use of Statins in Prevention: an Intervention Trial Evaluating Rosuvastatin (JUPITER), Anglo‐Scandinavian Cardiac Outcomes Trial (ASCOT), Cholesterol and Recurrent Events (CARE), and Pravastatin or Atorvastatin Evaluation and Infection Therapy–Thrombolysis in Myocardial Infarction (PROVE‐IT‐TIMI). The first two trials assessed statin therapy as primary prevention and the latter two trials as secondary prevention. Results of these trials showed statin therapy was effective for primary and secondary prevention. Samples that had been stored were genotyped for the 27 genetic risk variants. The results showed that the Polygenic Risk Score detected those at the highest risk for CAD. The GRS predicted cardiac events independent of traditional risk factors. The individuals ranked at the highest genetic risk were also the individuals receiving the highest proportion of statin therapy. The increased power of the GRS over conventional risk factors is evident from the observation that to prevent one cardiac event the number to be treated was decreased 3‐fold from that of conventional risk factors. Those with the highest genetic risk received the most benefit from statin therapy. The power of the GRS to stratify and select those at greatest risk was equally effective in both primary and secondary prevention. Another large clinical trial^30^ referred to as the West of Scotland Coronary Prevention Study (WOSCOPS) was analyzed utilizing 57 genetic risk variants. Statin therapy in the high genetic risk group was associated with a 44% reduction of cardiac events, versus only 24% in the intermediate and low‐risk genetic groups. The number needed to treat to prevent one cardiac event in the high genetic risk group was 13 individuals versus 38 in the intermediate and low genetic risk groups. These results demonstrate the greater power of genetic risk stratification for CAD over that of traditional risk factors.

Until recently, decreasing plasma cholesterol to prevent CAD primarily employed statins. Recently, a new armamentarium was introduced, the PCSK9 inhibitors, which act by removing LDL‐C from the plasma and are thus complementary to statins which inhibit the synthesis of cholesterol. Two recent randomized placebo‐controlled clinical trials evaluated the effect of PCSK9 inhibitors on cardiac events. The FOURIER (Further Cardiovascular Outcomes Research with PCSK9 inhibition in subjects with elevated risk), trial^31^ evaluated Evolocumab (PCSK9 inhibitor) in 11 953 patients. The risk for CAD was determined utilizing the polygenic risk score which categorized risk into high, intermediate, and low. There was a strong correlation between individuals with intermediate and high genetic risk for CAD and cardiac events with 1.23 and 1.65 hazard ratios respectively. Patients receiving Evolocumab therapy had a 13% relative reduction in the group stratified by conventional risk factors without high genetic risk and a 31% reduction in the high genetic risk group with or without conventional risk factors. Individuals with the highest genetic risk had the most benefit from lowering plasma LDL‐C due to Evolocumab and was independent of conventional risk factors.

The ODYSSEY (Evaluation of Cardiovascular outcomes after an acute coronary syndrome during treatment with Alirocumab) trial^32^ enrolled 11 953 individuals. Individuals with the highest GRS had the highest risk for CAD and Alirocumab was associated with a 37% reduction in cardiac events versus a 13% reduction in the group with the lowest GRS. These results confirm that the GRS is effective in identifying those at greatest risk who will receive the greatest benefit from lowering cholesterol.

The results of these studies are summarized in Table 1.

**TABLE 1:** Comparison of results using the PRS as a means to stratify risk for CAD

		NNT		RRR (%).		
	Study	High genetic risk	Low genetic risk	High genetic risk	Low genetic risk	Sample size
Statin therapy	Mega et al.	25	66	50	34	48 421
	WOSCOPS	13	38	44	24	10 456
PCSK9 inhibitors	Fourier			31	5	14 298
	ODYSSEY	17	64	37	13	11 953
Lifestyle changes	Khera et al.			46	45	55 685

*Note*: The clinical trials by Mega et al. and West of Scotland Coronary Prevention Study (WOSCOPS) evaluated the effect of statin therapy versus placebo in patients with Coronary Heart Disease (CHD). Genetic risk stratification identified the group at greatest risk, which was also the group that benefited most from statin therapy. The Fourier and ODYSSEY Clinical Trials evaluated the effect of PCSK9 inhibitors vs. placebo in patients with CHD. Genetic risk stratification identified the group at greatest risk, which was also the group that benefited the most from PCSK9 inhibitors. The study be Kera et al. evaluated the effect of a favorable diet versus an unfavorable diet. Genetic Risk Stratification identified the group at risk, which was also the group that benefited most from a favorable diet. NNT, the number needed to treat. RRR, the relative risk reduction. Sample size, the total number of patients studied.

**Prospective evaluation of polygenic risk score in large biobank populations**

Abraham et al evaluated the GRS in five prospective cohorts, 3 from the FINRISK group and 2 from the Framingham Heart Study for a combined sample size of 16 082 subjects. In this study, 49 310 risk variants for CAD derived from CARDIoGRAMplusC4D were genotyped from which a GRS was developed. These investigators observed those at the highest genetic risk with the highest GRS to be strongly associated with cardiac events independent of cardiac risk factors, including family history. The combination of the GRS with the result of the pooled cohort equations (derived from conventional risk factors) improved the 10‐year risk prediction for CAD events.^33^

Utilizing the UK Biobank, with a sample size of nearly 500 000, Inouye et al^34^ genotyped with 1.7 million genetic risk variants predisposing to CAD. The top 20% with the highest polygenic risk score had a 4‐fold increased risk of CAD. The power of the GRS to risk stratify for CAD was relatively independent of conventional risk factors. The investigators remarked that their use of 1.7 million genetic risk variants was superior to the previous studies utilizing 27, 57, or 49 310 genetic risk variants.^29^, ^30^, ^33^ A similar analysis was performed utilizing the UK biobank with a sample size of 388 978 individuals.^35^ The samples were genotyped for 6.6 million risk variants predisposing to CAD. The results showed that the top 8% with the highest GRS had a 3‐fold increased risk for CAD, and the top 0.5%, a 5‐fold increased risk for CAD. The investigators further analyzed the power of conventional risk factors vs the GRS. In the group with the highest GRS, only 20% had hypercholesterolemia, 35% a family history, and 28% had hypertension, showing GRS is superior to, and relatively independent, of conventional risk factors.

**Favorable lifestyle changes significantly reduce genetic risk for CAD**

Genetic risk stratification for CAD has been evaluated in over 1 million individuals and shown to be superior to Risk Scores based on conventional risk factors. The next important question is whether one can prospectively reduce the genetic risk for CAD. An analysis of four prospective cohorts was performed by Khera et al^36^ involving a sample size of 55 685 individuals. The analysis showed those with a high GRS for CAD (top 20%) had a 90% higher risk of cardiac events than the remainder. The objective of the study was to compare the incidence of cardiac events in individuals with a healthy lifestyle (no current smoking, no obesity, regular physical activity, and a healthy diet) to that of an unhealthy lifestyle (at least 2 unfavorable features). In the group with the highest GRS and a favorable lifestyle, there were 46% fewer cardiac events than those with an unfavorable lifestyle.

Utilizing a large sample size of 468 095 individuals from the UK Biobank, Tikkanen et al^37^ assessed the effect of physical activity on the risk of cardiac events stratified using the GRS. The physical activity performed was that of the handgrip for 3 s and cardiac respiratory fitness was assessed by oxygen consumption performed while exercising on a stationary bike. In the highest genetic risk group, those having the most benefit from cardiorespiratory fitness exhibited a 49% lower risk for CAD.

**Limitations to the Current Polygenic Risk Score**

The polygenic risk score for CAD is based on genetic risk variants discovered by GWAS performed on populations that are 77% of European descent.^38^ It is reasonable to expect that different ethnic groups may have developed variants unique to these groups. The large migration out of Africa about 100 000 years ago predisposed to different genetic adaptation and evolution in response to the different environments of Asia, Europe, and ultimately the Americas. The population remaining in Africa would continue to admix and generate new variants while those in different areas of the planet would be isolated from these variants. The migratory population might develop, perhaps more slowly, variants unique to their adaptation. Iribarren et al^39^ performed a study in populations of African (*n* = 2089), Latino (*n* = 4349), and East Asian (*n* = 4804) ancestry. The GRS was derived from 51 genetic risk variants known to predispose to CAD. These risk variants were determined primarily by individuals of European descent.^38^ The results of the GRS screen for risk of CAD were compared to that of the Framingham risk score (FRS) to estimate the 10 year CAD risk. They observed that the polygenic risk score reclassified about 10% of the population in the intermediate‐risk group of the FRS in both Latinos and African Americans, but less in East Asians. The investigators concluded the GRS was an improvement over conventional risk factors but did not recommend adoption for routine clinical management.

Recognizing the potential for loss of power of the polygenic risk score in different ethnic groups. Dikilitus et al^40^ performed a study on individuals of European ancestry, African ancestry, and Hispanic ethnicity. The sample size included 45 645 individuals of European ancestry, 7597 of African ancestry, and 2493 of Hispanic ethnicity. Each of the populations was genotyped with an array containing 1.7 million and the other 6.6 million genetic risk variants. The polygenic risk score was strongly associated with risk for CAD in all 3 cohorts. The hazard ratio's per SD increase was 1.53, 1.53, and 1.27 for the incidence of CAD in European ancestry, Hispanic ethnicity, and African American individuals, respectively. The hazard ratios were comparable in the European and Hispanic cohorts but significantly attenuated in individuals of African American descent. These results suggest the polygenic risk score, despite being derived from European ancestry, was effective in risk stratifying for CAD in all three populations, but less powerful in African Americans.

The South Asian population that includes primarily India, Pakistan, Bangladesh, Bhutan, and Sri Lanka comprise about 1.8 billion people. East Asians are thought to have a high incidence of heart disease. Wang et al^41^ pursued studies on South Asians (*n* = 7244) who participated in the UK biobank, together with separate populations from Bangladesh and India. They utilized a polygenic risk score based on genotyping of 6 630 150 genetic risk variants derived primarily from individuals of European descent. The polygenic risk score was effective in stratifying risk for CAD across South Asians from all three populations. They noted a 3.22 to 3.91 fold increase in risk upon comparing the highest to the lowest quintiles across three independent cohorts. This indicated that the polygenic risk score is effective across different ethnic groups. Results were somewhat attenuated compared to the results obtained upon analyzing participants of European ancestry where the odds ratio per SD increment was 1.72 compared with 1.58 to 1.66 observed in the South Asian populations. It is of note that about 5% of the Indian cohort inherited about triple the normal risk based on the polygenic variation. It is important to also note that these individuals could not be reliably identified from the remainder of the population based on conventional risk factors. About a third of the patients would be reclassified for risk of CAD by the GRS compared to the conventional Framingham Risk Score. Risk stratification for CAD gave an odds ratio per standard deviation of 1.58 in South Asian UK biobank participants and 1.60 in the Bangladesh study. Risk stratification for CAD showed the risk for CAD in the top 5% of the GRS had odds ratios of 4.16, 2.46, and 3.22 in the South Asian UK biobank, Bangladesh, and Indian cohorts, respectively. The power of the polygenic risk score in a particular ethnic group may be enhanced by including genetic risk variants derived from the population under consideration.

**Embracement of the GRS into clinical application for primary prevention of CAD**

**FIGURE 2:**
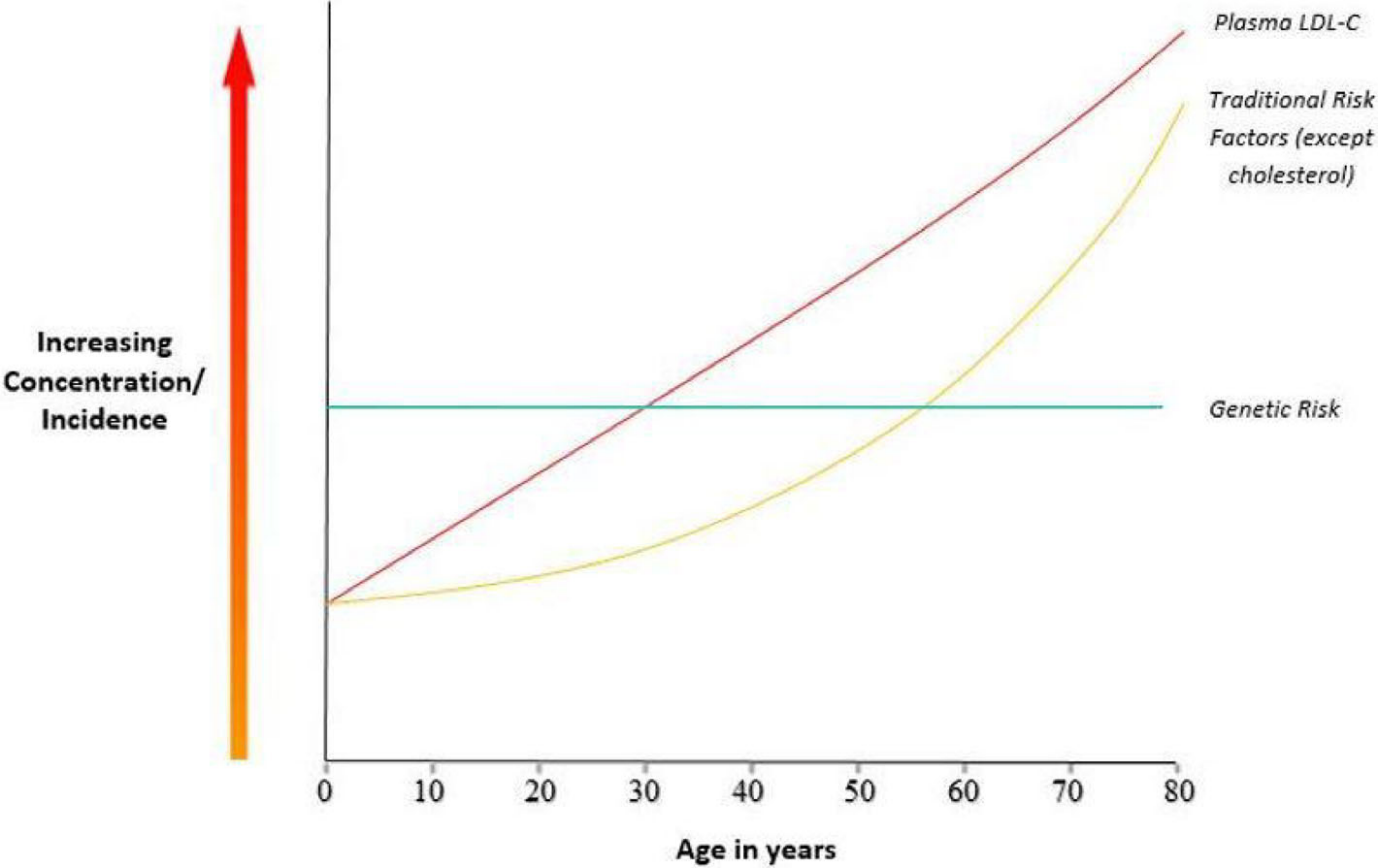
Genetic versus traditional risk stratification for coronary artery disease (CAD). Early primary prevention is limited based on traditional risk factors as shown in this figure. Traditional risk factors such as age, hypertension, or diabetes are infrequent until the 50's or 60's. Cholesterol (red) is an exception which increases early in life and the risk for CAD doubles every 10 years. In contrast, the genetic risk score for CAD (blue) is independent of age and remains the same throughout life. The genetic risk obtainable at any time after birth provides a major advantage enabling one to predict risk for CAD early in life. This could be a paradigm shift for the implementation of primary prevention

The hope of halting the pandemic of coronary artery disease is greatest if we can institute primary prevention to prevent diseases. A major barrier to primary prevention is detecting those at risk early in the asymptomatic stage of CAD. The peak incidence of the clinical manifestations of CAD in the male is around 58 years and around 68 years in the female. Initiating early primary prevention in the female may be very effective in the fifth decade and probably less effective in males. The conventional risk factors, such as hypertension, diabetes, become evident in most people only after the sixth and seventh decades. Plasma cholesterol is the only conventional risk factor that is significantly elevated early in life^42^, ^43^ as shown in Figure 2. The other conventional factors in the third and fourth decade are seldom present and cannot be used to initiate primary prevention. Given plasma cholesterol is elevated in most Americans,^27^ if not in most Westerners in the third and fourth decade of life, one might argue, why not treat everyone? The therapy to decrease plasma cholesterol is accessible and inexpensive. The side effects are minimal but the cost would still be significant to treat everyone with therapies such as statin and even more so if one uses PCSK9 inhibitors. The third factor to be considered is that epidemiologists^27^ have long recognized that only about 50% of the population will experience a cardiac event even if they live the normal mean lifespan. In an era where medicine is supposed to be more precise and cost‐effective, one would prefer initiating primary prevention in those at risk for the disease.

**FIGURE 3:**
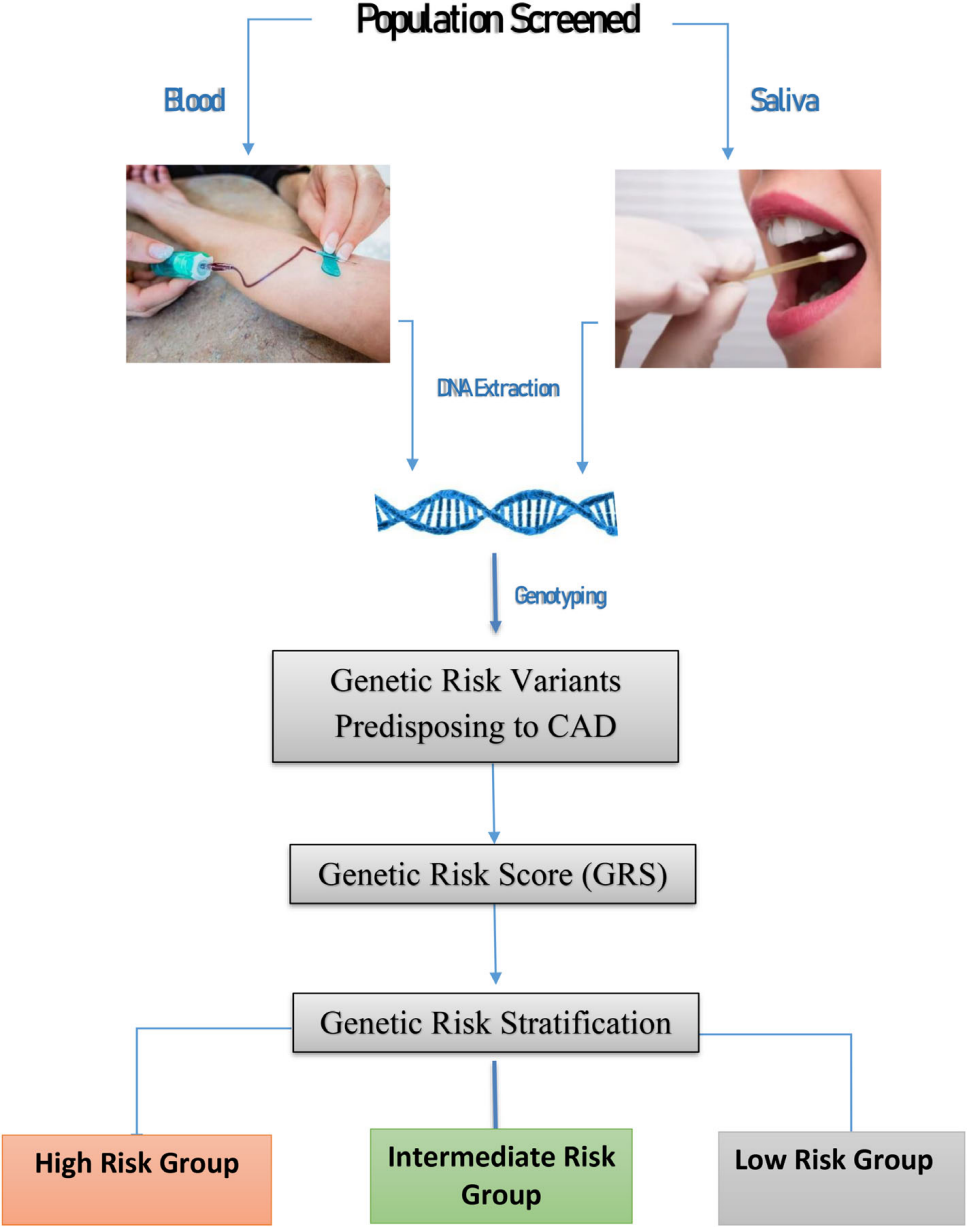
Genetic risk screening for coronary artery disease (CAD). The sample for DNA analysis can be obtained either from saliva or blood. The DNA will be genotyped for the genetic risk variants predisposing to CAD. The polygenic risk score is calculated as a single number. Based on the GRS, the patients are stratified into three separate groups; high, intermediate, and low risk

The polygenic risk score is superior to conventional risk factors and clinical trials have shown genetic risk to be markedly reduced by statin therapy, PCSK9 inhibitors, and lifestyle changes. The age dependency of conventional risk factors does not apply to the polygenic risk score, since one's genetic risk is determined and randomly assigned at conception and does not change in one's lifetime. Thus, the GRS can be determined early in life and serve as the basis to initiate primary prevention of CAD as illustrated in Figure 3. While the polygenic risk score has not yet been incorporated into the cardiology clinical guidelines, the recent revision of the clinical guidelines offers some flexibility by proposing the use of enhancers to stratify for risk. Genetic Risk Stratification as proposed in Figure 3 could be incorporated as an enhancer similar to the recent embracement of the calcium score. In a recent study^44^ by Riveros‐Mckay et al, the investigators integrated a PRS with the Pooled Cohort Equation (PCE) used by the U.S. guidelines. They observed 10% of CAD cases were misclassified as low risk by PCE which were correctly classified as high risk by the integrated PRS and PCE. They up‐classified 7% of the population (7.4 million individuals) to high risk, qualifying them for statin prevention which they estimate would save 12 000 lives over 5 years. The cost of the genotyping and the development of the polygenic risk score would be expected to be in line with other blood tests. The cost is often stated to be between $50 and $100,^34^ however, a commercial company is currently charging $350. In one's lifetime, this test would only have to be conducted once and, as the demand increases, one would expect the cost to be in the range of $100–$200 if not less. The application of the polygenic risk score to males in their 20's and 30's or females in their 40's would be expected to transform the primary prevention of the world's number one killer, CAD. Primary prevention would be expected to prevent the progression of coronary atherosclerosis and subsequent clinical sequelae.
